# Malaria micro-stratification using routine surveillance data in Western Kenya

**DOI:** 10.1186/s12936-020-03529-6

**Published:** 2021-01-07

**Authors:** Victor A. Alegana, Laurissa Suiyanka, Peter M. Macharia, Grace Ikahu-Muchangi, Robert W. Snow

**Affiliations:** 1 Population Health Unit, Kenya Medical Research Institute-Wellcome Trust Research Programme, P.O. Box 43640-00100, Nairobi, Kenya; 2 Geography and Environmental Science, University of Southampton, Southampton, SO17 1BJ UK; 3 Faculty of Science and Technology, Lancaster University, Lancaster, LAI 4YW UK; 4 National Malaria Control Programme, Ministry of Health, P.O Box 30016-00100, Nairobi, Kenya; 5 Centre for Tropical Medicine and Global Health, Nuffield Department of Clinical Medicine, University of Oxford, Oxford, OX3 7LJ UK

**Keywords:** Malaria, Routine data, Test positivity rate

## Abstract

**Background:**

There is an increasing need for finer spatial resolution data on malaria risk to provide micro-stratification to guide sub-national strategic plans. Here, spatial-statistical techniques are used to exploit routine data to depict sub-national heterogeneities in test positivity rate (TPR) for malaria among patients attending health facilities in Kenya.

**Methods:**

Routine data from health facilities (*n* = 1804) representing all ages over 24 months (2018–2019) were assembled across 8 counties (62 sub-counties) in Western Kenya. Statistical model-based approaches were used to quantify heterogeneities in TPR and uncertainty at fine spatial resolution adjusting for missingness, population distribution, spatial data structure, month, and type of health facility.

**Results:**

The overall monthly reporting rate was 78.7% (IQR 75.0–100.0) and public-based health facilities were more likely than private facilities to report ≥ 12 months (OR 5.7, 95% CI 4.3–7.5). There was marked heterogeneity in population-weighted TPR with sub-counties in the north of the lake-endemic region exhibiting the highest rates (exceedance probability > 70% with 90% certainty) where approximately 2.7 million (28.5%) people reside. At micro-level the lowest rates were in 14 sub-counties (exceedance probability < 30% with 90% certainty) where approximately 2.2 million (23.1%) people lived and indoor residual spraying had been conducted since 2017.

**Conclusion:**

The value of routine health data on TPR can be enhanced when adjusting for underlying population and spatial structures of the data, highlighting small-scale heterogeneities in malaria risk often masked in broad national stratifications. Future research should aim at relating these heterogeneities in TPR with traditional community-level prevalence to improve tailoring malaria control activities at sub-national levels.

## Background

The highest public health burden posed by infection with *Plasmodium falciparum* continues to be borne by countries in sub-Saharan Africa (SSA) [1]. Infection prevalence and disease risks remain unevenly distributed between and within countries [2, 3]. This spatial heterogeneity requires strategies that facilitate targeting of limited resources for malaria control, as outlined in WHO’s Global Technical Strategy (GTS) for malaria [4] and the High Burden-High Impact (HBHI) initiative [5]. Current national malaria strategic plans in SSA use a variety of metrics to depict sub-national variations in malaria risk ranging from modelled community-based parasite prevalence to crude estimates of clinical incidence from routine data [6]. The main challenge for National Malaria Control Programmes (NMCPs) is in using all available data, effectively, to provide robust malaria risk maps that can guide micro-stratification.

Malaria routine data from District Health Information System 2 (DHIS2) summarized as test positivity rate (TPR) among patients attending health facilities is a simple metric, providing a means for micro-stratification and targeted responses [7–13]. Compared to cross-sectional community-based surveys of infection prevalence, TPR is more ubiquitous in time and space because data are collected continuously and across all treatment facilities in a locality.

Traditionally, NMCPs define TPR as a ratio of aggregated number of confirmed cases over parasitological tests undertaken within a single administrative unit. Such an approach does not adjust for: (a) the spatial and temporal heterogeneities in the data at a more granular scale; (b) the populations who would use health facilities at the borders of administrative units; or, (c) missingness of the reported data by health facility. Importantly, NMCPs rarely consider uncertainty related to indicator estimation, which are important metrics for decision-making when choosing between malaria strategies [12, 14–18].

Here, the aim was to provide an example of quantifying the spatial heterogeneities in TPR using a Bayesian model-based framework [19, 20] adjusting for data missingness, spatio-temporal dependencies and population density at fine-scale to guide malaria micro-stratification in Western Kenya.

## Methods

### Study setting

**Fig. 1:**
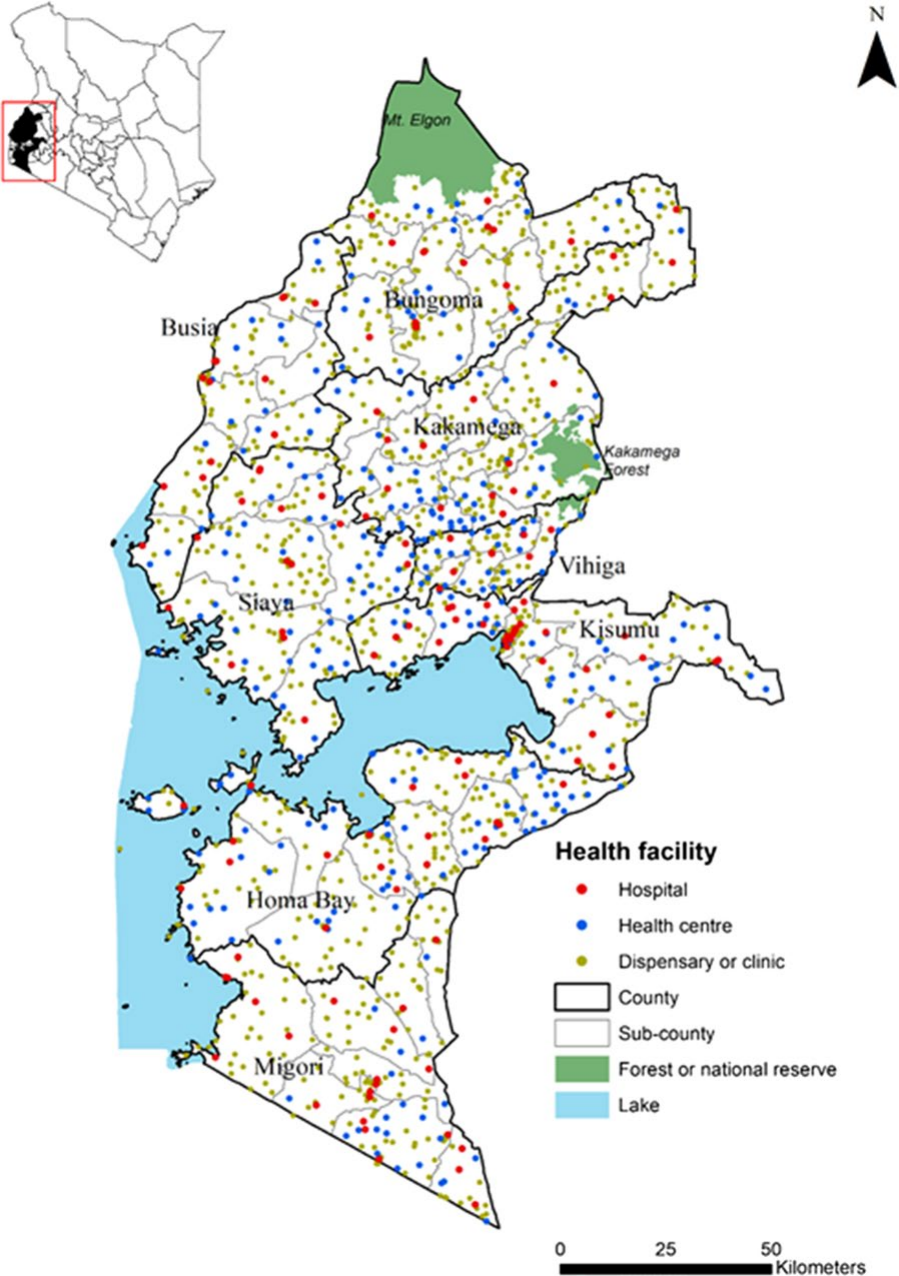
Distribution of health facilities in the study area

The present study used routine health facility data from 8 counties in Western Kenya: Bungoma, Busia, Homa Bay, Kakamega, Kisumu, Migori, Siaya, and Vihiga. These counties represent devolved administrative units responsible for making sub-national decisions on the provision of health care, including malaria, and are administratively sub-divided into 62 sub-counties (Fig. 1). The NMCP provides overall national malaria policies, strategic direction and coordinates bi-lateral and multi-lateral support for national malaria control while counties are expected to adapt national policies to their local epidemiological context [21].

The 8 Lake-endemic counties (Fig. 1) cover 19.4% (9.4 million people) of Kenya’s population [22]. The area experiences two rainy seasons, March to May and October to December; malaria transmission is intense throughout the year with community-based *Plasmodium falciparum* prevalence among children exceeding 30% in 2009 [23], and with the highest rates of malaria transmission in Kenya in 2015 [18]. Transmission is maintained by high biting rates from local vector populations including *Anopheles funestus *sensu stricto (s.s), *Anopheles arabiensis* and *Anopheles gambiae *s.s. [24, 25].

Between 2016 and 2019, 1.1 million long-lasting insecticide-treated nets were distributed routinely (antenatal clinic clients) across the 8 counties. Since 2017, there have been 3 rounds of indoor residual spraying (IRS) in Homa Bay and Migori counties using Actellic 300 CS and SumiShield 50 WG [26]. In September 2019, 23 sub-counties in Western Kenya were randomly allocated to receive the Food and Drug Administration (FDA)-approved RTS,S/AS01 (RTS,S) vaccine and form part of an ongoing evaluation of safety and effectiveness [27].

### Routine malaria data from DHIS2

An aggregate of monthly outpatient malaria cases representing presentations among all ages to public and private health facilities was obtained from the DHIS2. Data were assembled for 24 months from January 2018 to December 2019. DHIS2 is the electronic routine health data platform for reporting, analysing and disseminating data for health programmes, piloted in 2010 and rolled out national-wide in Kenya in 2011 [28, 29]. Health facilities comprised of level 4 or level 5 (hospitals), level 3 (health centres) and levels 2 and 1 (primary care facilities or dispensaries) [30].

Recent evidence shows that over 90% of suspected malaria cases are subjected to a malaria parasitological test in Western Kenya [31]. Malaria rapid diagnostic tests (RDTs) were introduced to scale-up fever testing of all age groups in 2012 in Kenya [32]. The focus of the present analysis was on the monthly aggregated number of patients suspected for malaria (the denominator) and the number of cases of positive RDT or blood slide-confirmed malaria cases (the numerator), excluding follow-up visits and referrals, resulting in a TPR. It was not possible to identify the 10% fevers clinically diagnosed from the aggregated monthly data. Thus, the definition of TPR is not the strict definition of fever test positivity rates used as a historical metric of malaria risk that aimed to test all fevers [33, 34], but a suspected malaria TPR, based on service provider perceptions of probable malaria.

### Population data

Fine spatial resolution, 1-km gridded population data for Western Kenya was derived using the 2019 national census data available at sub-county levels [22], and distributions of populations at enumeration area (EA) levels used during the 2009 census as input data. Standardized dasymetric distributions were used to allocate population density weights using a random forest (RF) model [35]. The modelled EA population distribution was projected to 2019 using 2009–2019 inter-censual growth rates and matched to 2019 sub-county census population estimates. Population adjustments were modelled based on land cover using the RF model to provide a continuous 1-km gridded estimate of population in 2019 map (Additional file 1).

### Data pre-processing and geo-referencing

DHIS2 data completeness was checked based on the number of facility monthly reports recorded out of the expected number of facility-month reports. The expected number of reports was calculated from the universe of public and private facilities in the 62 sub-counties. The master health facility list of operational facilities, had been geocoded to provide spatial locations, described elsewhere [23, 36].

### A hierarchical space–time geostatistical analysis of TPR

The geographic coordinates of the health facility combined with data indexed in time (month) allowed the prediction of TPR using a hierarchical Bayesian space–time modelling context adjusting for three broad levels of service provision (hospitals, health centres and dispensaries or clinics). The interest was to define the underlying spatial–temporal process of TPR. Since a universe of all facilities was available and geocoded, the space–time analysis aimed at predicting TPR in space at 1 km × 1 km pixels to match population distribution. This scale was used, rather than county or sub-county, to allow for the fact that facilities are located on administrative boundaries serving more than one administrative population and assumes that if a facility was located at each grid it would have TPR properties to those most proximal and temporal (month) to existing, reporting facilities. Fine-scale (1 km × 1 km) TPR predictions were then aggregated as the average, population-weighted area estimates at the sub-county level.

To predict gridded estimates of TPR, the methodology exploits the spatial and temporal autocorrelation in outpatient case counts to predict the missing or unsampled values as weighted linear combinations of the data points close in space–time. Thus, using the health facility spatial location ${s}_{i}\left({s}_{i}=1,...,n\right)$, the corresponding number of people visiting the health facility suspected with malaria $N\left({s}_{i},t\right)$, month (time) $t\left(t=1,...,T\right)$ and the number confirmed malaria cases $y\left({s}_{i},t\right)$, the modelling framework translates the discretised observations to a prediction of TPR. The important aspect of hierarchical Bayesian formulation is linking the observational data model to latent processes (the spatio-temporal process and the parameters). A binomial likelihood was used (data likelihood), combined with prior information containing uncertainty in the data generating process resulting in a posterior probability distribution. The data likelihood function for observational data given the linear predictor $\eta \left(s,t\right)$ was defined as:

$$y\left( {s,t} \right)|\eta \left( {s,t} \right)\sim Binomial\left( {N\left( {s,t} \right),P\left( {s,t} \right)} \right)$$

where $\eta \left( {s,t} \right) = logit\left( {P\left( {s,t} \right)} \right)$. The spatio-temporal process, defined on the linear predictor as:

$$\eta \left( {s,t} \right) = \alpha_{0} + X\left( {s,t} \right)^{^{\prime}} \beta + w\left( {s,t} \right) + \gamma_{s} \left( t \right) + e\left( {s,t} \right)$$

where ${\alpha }_{0}$ is an intercept and for a generic location s, $X(s,t)$ is a set of covariates associated with health facility (the type of facility type and year) and $\beta$ are the corresponding regression parameters. $w(s,t)$ is a mean-zero spatio-temporal process and $e(s,t)$ are $i.i.dN\left(0,{\sigma }_{e}^{2}\right)$ and independent of other processes. The error term $e(s,t)$ is the residual adjustment to the spatio-temporal explanation. With $t=\mathrm{1,2},...T$, $\gamma \left(t\right)$ represent monthly variables adjusting for seasonality specified using first-order random walk. Missing data were imputed in space–time adjusting for facility type in a similar way to other data points.

Modelling was implemented using the Integrated Nested Laplace approximation (INLA) R-statistical package [37]. R-INLA uses both analytical approximation and numerical integration to perform approximate Bayesian inference for the class of latent Gaussian models, such as the spatio-temporal models [38]. The geostatistical implementation in R-INLA was implemented via the space–time stochastic partial differential equation (SPDE) approach [39]. The Bayesian specification was completed by assigning prior distribution for parameters of the random walk using the penalized complexity prior [40], SPDE, and fixed effect (flat priors) (Additional file 2).

### Micro-stratification using exceedance probability

Micro-stratification within counties is a priority for the county Ministries of Health, to set priorities for malaria control investment. However, a degree of certainty is necessary to set priorities [16, 17]. As such, exceedance probabilistic methodology [14, 15] was used on the fitted population-weighted model for TPR. This probabilistic estimate identified locations where ${p}_{c}\left(s,t\right)=P\{\eta (s,t)>l\}$ with $l$ as the threshold level of interest. A threshold of > 70% population weighted TPR represented high burden sub-counties (or the 10% sub-counties with highest TPR), while < 30% represented sub-counties with low malaria risk. In previous studied 30% TPR was associated with low malaria prevalence estimated from community survey data [41, 42]. Thus, areas where ${p}_{c}\left(s,t\right)$ was closer in value to 100%, indicated the likelihood of location to be above the threshold $l$. Conversely, when ${p}_{c}\left(s,t\right)$ value was close to 0% indicated an increased likelihood of being below the threshold. For ${p}_{c}\left(s,t\right)$ equal to 50% corresponded to sub-counties with the highest uncertainty, with an equal probability below or above the threshold $l$.

### Model validation procedures

Cross-validation techniques were used to evaluate the predictive performance of the model. This was based on a 20% sub-set of data selected randomly and used in the computation of prediction error metrics namely: the mean absolute error, the mean prediction error (MPE), mean absolute error (MAE), the root mean square error (RMSE), and a Pearson’s product-moment correlation coefficient that quantified the association between observed and predicted values.

## Results

### Data coverage and reporting rate

**Fig. 2:**
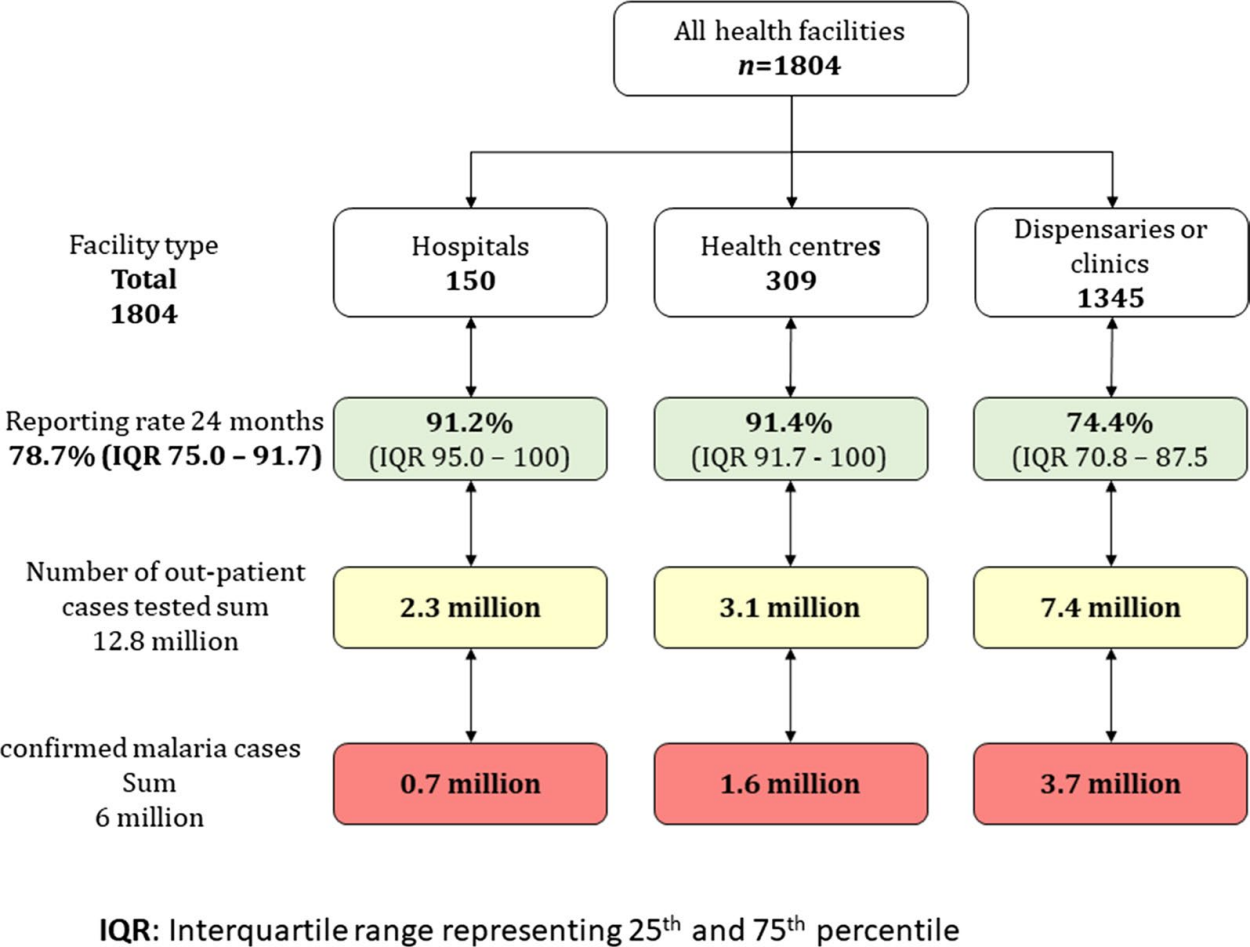
Malaria data summary for the 8 counties

Figure 2 provides a summary of assembled data by the type of health facility among the expected 1804, including 150 hospitals, 309 health centres and 1345 dispensaries and clinics. Only 160 health facilities (8.9%) did not report any data for the 24 months, with 147 being the lowest level of facility (dispensaries or clinics). The overall monthly reporting rate for the data period was 78.7% (IQR 75.0–100.0); 1339 (74.2%) facilities reported data for 18/24 or more months, 264 (14.6%) reported for at least 12 months, and 41 (2.3%) facilities reported data to the DHIS2 for 6 months or less. Analysis suggested that public-based health facilities were more likely than private facilities to report ≥ 12 months (OR 5.7, 95% CI 4.3–7.5), and dispensaries or clinics had lower odds of reporting ≥ 12 months (OR 0.4, 0.3–0.7). Lastly, there was no difference in TPR by age in the DHIS2 (for under-5 years 47.4% 95% CI 45.9–48.9 compared to the over-5 years 47.8% 95% CI 46.2–49.2, respectively). Therefore, all subsequent analysis of TPR was aggregated for all ages.

A total of 6.0 million outpatient malaria cases were confirmed at health facilities among 12.8 million suspected malaria outpatient cases over 24 months. The 24-month mean for hospitals was 688 confirmed cases compared to 309 cases at primary-level facilities over the 24 months. The number of confirmed cases varied by month ranging from 0.2 million confirmed cases among 0.6 million suspected cases in December and highest in July, 0.8 million confirmed among 1.7 million suspected.

### TPR model sensitivity analysis

Model validation was assessed using the MAE as well as an assessment of prediction performance based on the 20% validation sample. The MPE summarizing the difference between predicted and observed values was 0.01 while the MAE was 0.12 and the RMSE 0.2. Pearson’s correlation between observed and predicted values was 0.64 (p < 0.0001). The analysis of residuals showed minimum spatial autocorrelation after modelling as depicted in the semi-variogram of the residuals. The residual variogram was within the 95% interval suggesting that the spatial structure in the data was accounted for by the space–time modelling (Additional file 2). The model spatial range was approximately 20.7 km (95% Bayesian credible interval in km 16.4–25.7). Additional file 2: Table S2 lists the posterior summaries of the parameters of space–time modelling representing the fixed effects and the temporal and spatial parameters. From these parameters, adjusting for the year of data point and facility type (level) was important in the model estimation at the 95% Bayesian credible interval.

### Spatial heterogeneity in TPR at sub-county level

**Fig. 3:**
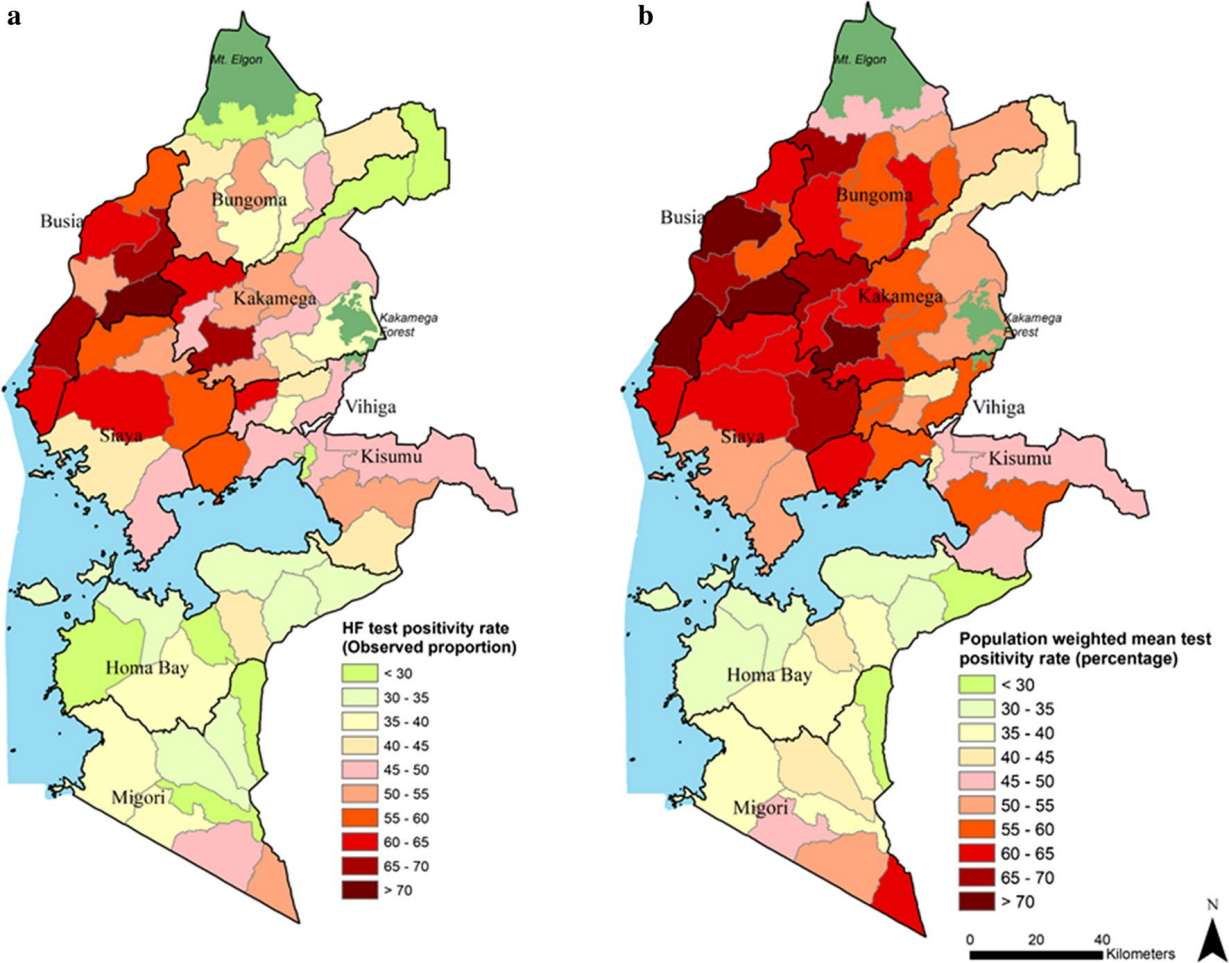
Maps of crude and population-weighted modelled test positivity rate

**Fig. 4:**
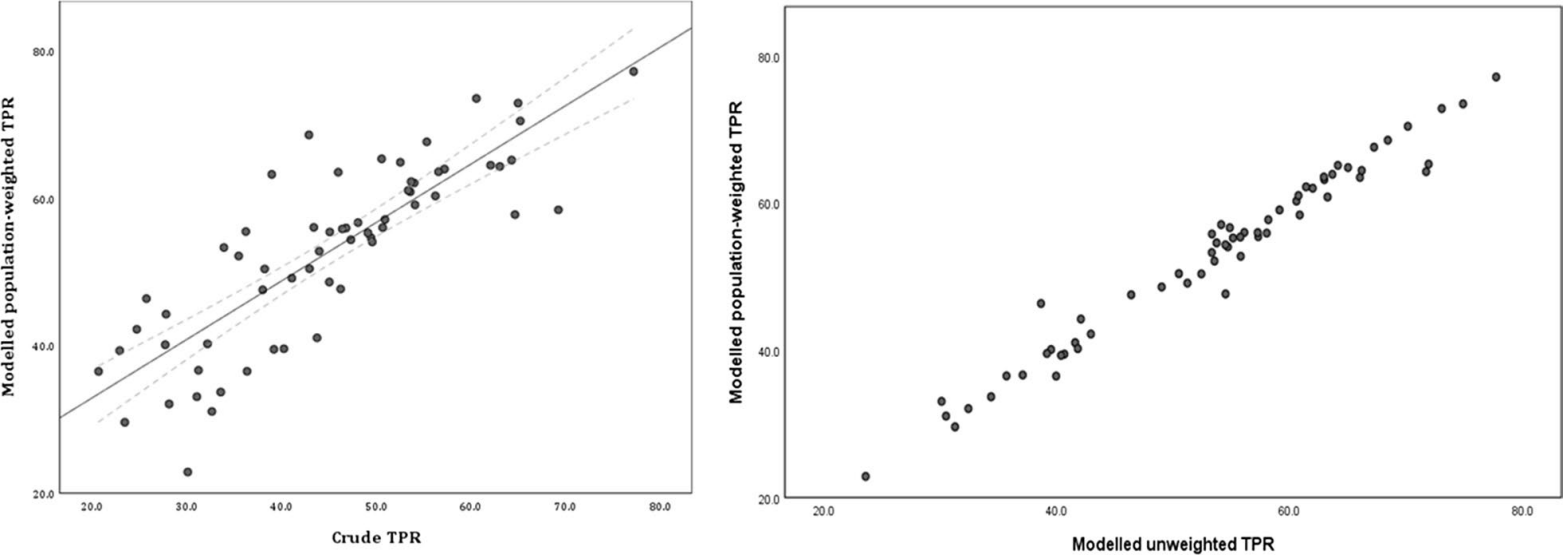
Comparison of crude TPR with modelled estimate

Figure 3a shows the crude aggregated TPR, compared to Fig. 3b which shows the modelled population weighted TPR for each of the 62 sub-counties. Both crude and adjusted sub-county TPRs highlight the marked heterogeneity across the region. The crude estimates, however, do not adjust for population density or missingness of the uncertainty in data. Figure 4a shows the differences between the crude TPR to the modelled population-weighted estimates while Fig. 4b shows the difference when compare to the unweighted mean. Population weighting adjusts the modelled TPR estimates within the sub-county based on population distribution. There were differences between crude and modelled estimates particularly in the sub-counties in the north of the Lake, e.g., Bungoma county. Higher modelled population-weighted TPR areas were also located in these northern sub-counties. At a county level, the highest mean predicted TPR was in Busia county, mean 70.6% (95% Bayesian credible interval 68.1–72.8%); 6 sub-counties in Busia (Bunyala, Butula, Samia, Matayos and Teso South) and Kakamega (Butere) had TRP greater than 70% (Additional file 3). Homa Bay county had the lowest population weighted TPR 33.2% (30.4–36.0%). Rachuonyo East sub-county in Homa Bay was the lowest 23.6% (22.1–25.1%).

### Population at risk and micro-stratification at sub-county level

**Fig. 5:**
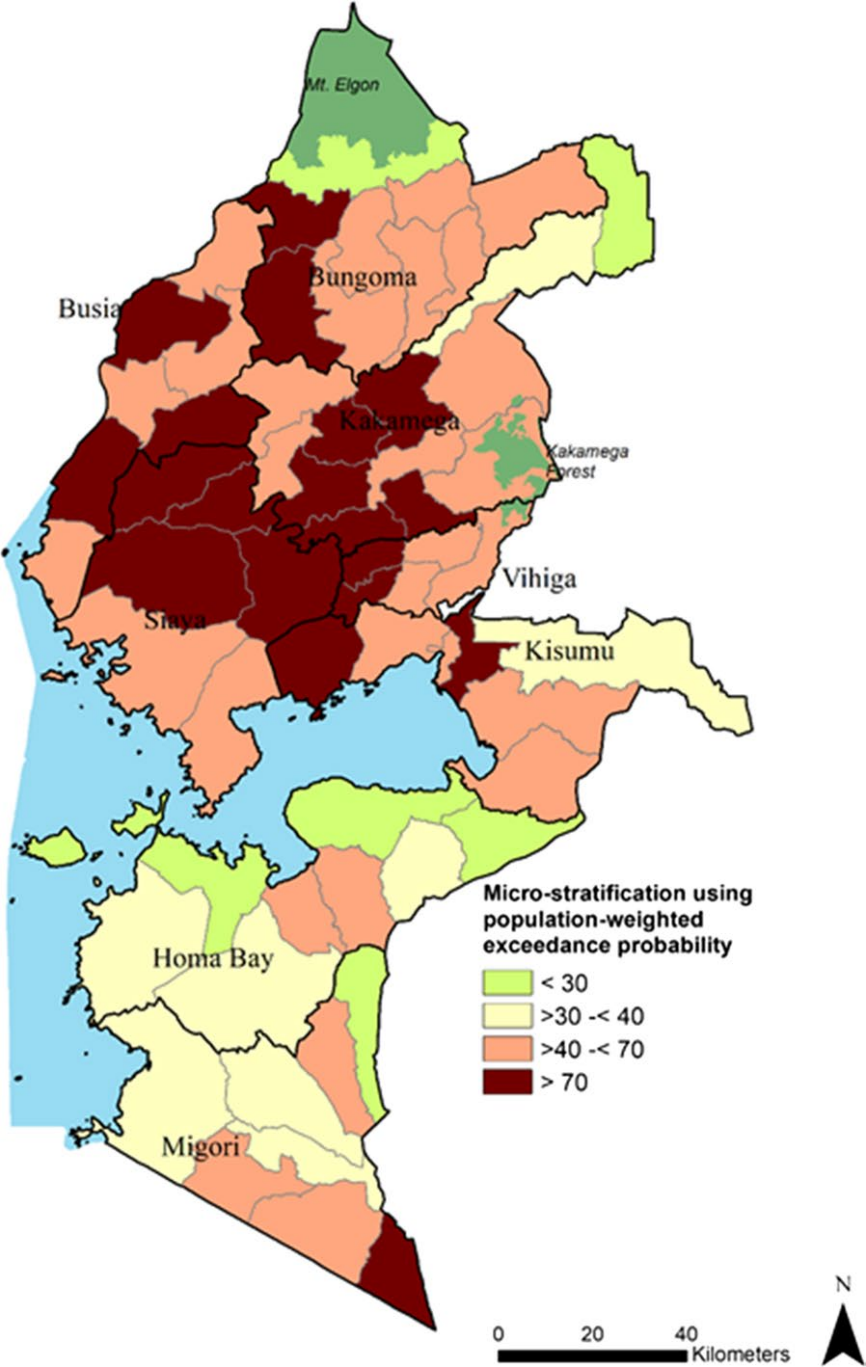
Map of exceedance probability

Of the 9.4 million residents of Western Kenya in 2019, 2.7 million (28.5%) lived in 19 sub-counties where the probability of TPR exceeded 70% at 10% chance of a Type I error occurring (Fig. 5). These were predominantly in the north of Lake Victoria and for the two sub-counties in Migori. Some 3.1 million (32.6%) lived in areas where TPR was likely to be ≥ 40% and < 70% (19 sub-counties), and 1.5 million (15.8%) lived in 10 sub-counties where TPR was ≥ 30% but less than 40%. Finally, approximately 2.2 million (23.1%) lived in 14 sub-counties where TPR was likely to be < 30% at 90% certainty (Additional file 3) corresponding to low-risk sub-counties where IRS was recently implemented.

## Discussion

Routine data for micro-stratification in stable, malaria-endemic settings should increasingly form the basis for tailoring malaria control and monitoring the impact of intervention(s) [6, 8]. Here, a geostatistical approach was applied to routine data from 8 counties of Western Kenya to explore heterogeneities in TPR to inform a micro-stratification at the sub-county level (*n* = 62). These outputs have immediate potential to enhance the capacity of decision-makers for malaria control within the devolved national structure. The Western Kenya region has high coverage of health facilities congruent to population density and with good reporting rates (79%) of malaria outpatient data to the national surveillance system (DHIS2). However, crude estimates of TPR data (Fig. 3a) do not account for the underlying spatio-temporal structure of the data, missingness and the underlying heterogeneous population distributions within each sub-county (Figs. 3b, 4).

There was marked heterogeneity in TPR with sub-counties in the north of the Lake exhibiting the highest TPR (exceedance probability > 70% with 90% certainty) where approximately 2.7 million (28.5%) people reside. The regions with the highest malaria burden would require concerted effort to increase vector control and other interventions to reduce transmission and consequent morbidity. Evaluating the probability of TPR exceeding a certain threshold promotes a policy-relevant dialogue on uncertainties related to estimates. For example, if one was to include chemo-prophylactic initiatives [43] to accelerate a reduction in transmission, the targeting of these interventions and the added costs would require some level of certainty. The Bayesian credible intervals presented for TPR account for the 21% missing data and account for the uncertainty introduced by the need to predict these missing data in time. Importantly, under-reporting at health facility level contributes to the underestimation of TPR when using crude estimates. Thus, modelling adjusts estimates of TPR to a likely average accounting for the underlying population heterogeneities. In Kenya, national stratification was based on a threshold of parasite prevalence without further consideration of related uncertainty [23].

Carefully assembled TPR results also serve as a means to track the impact of malaria interventions. For example, the effect of IRS in the two counties of Homa Bay and Migori where 3 rounds of IRS had been implemented since 2017 all showed considerably lower predicted TPR than neighbouring sub-counties. Importantly, the two sub-counties in Migori (Kuria West and Kuria East) where IRS was not implemented had much higher population-weighted, adjusted TPR values between 2018–19, 60.8% (59.0–62.6%) and 54.5% (52.7–56.3%), respectively. Future applications of these routine data might include the possible impact of the pilot RTS,S vaccine programme. However, it is notable that aggregated DHIS2 do not currently allow for finer age-structured data beyond under and above 5 years of age which would limit a closer understanding of vaccine effectiveness among children < 2 years.

Combining metrics from routine data (DHIS2) with community parasite prevalence could potentially improve estimates of disease burden at the population level [44–46]. The underlying assumption is that the spatio-temporal correlation in TPR is usually driven by the underlying PR spatio-temporal structure. This requires further investigation over large regions and possible interaction with malaria co-infections. However, the immediate application of such a hybrid approach is dependent upon a better understanding of the relationship between TPR to PR at varying endemicity, which is not always linear [41, 42].

The estimation of TPR is dependent on reporting completeness, data quality (including diagnosis) and malaria treatment-seeking behaviour [1, 9]. Although prevalence of testing in the 8 counties was > 90%, the aggregated monthly data (denominator) did not distinguish between clinically diagnosed malaria and confirmed malaria. As previously observed these differences are not recorded consistently at the facility level or when submitted to the DHIS2 [47]. The 2015 Kenya Malaria Indicator Survey (KMIS) did not have adequate sampling at the sub-county levels (or lower) and across all ages to adjust for malaria treatment-seeking behaviour in present study. Fever treatment is usually assessed for children under the age of 5 years only in survey data. Therefore, future studies could improve estimation of TPR at micro-scale using empirical data on treatment-seeking behaviour across all age groups.

The analysis presented here was limited to 2-year time-series data and could potentially be improved by the inclusion of longer space–time data sets to extract long-term trends [48, 49]. However, the data before 2018 were influenced by nationwide medical staff strikes [50]. For the period considered (2018–2019), data from the lower-tier facilities were less likely to be complete compared to hospitals and suggests that the quality of data from these facilities remains inadequate [9], but can be improved by increased training of health workers and health records officers. There could be biases introduced due to the type of diagnosis at the facility level by using RDT or microscopy with varying sensitivities [51, 52]. RDTs are the most common diagnostic tools at the lower tier-facilities without a laboratory technician. However, no information was recorded on the type of RDT used. For microscopy, information on the quality of slide and reading was unavailable. The quality of diagnosis was not taken into account at the facility levels or the differences in fever testing rates, which is only possible through direct observational audits [12, 53, 54]. Finally, the quality of DHIS2 documentation is known to vary [47], and the reliability of individual records cannot be quantified without substantial health facility audits.

## Conclusion

Adjusting for population distributions, data missingness and building in statistical uncertainty can improve the value of routine data for malaria micro-stratification. These approaches can identify impacts of local-scale vector control and allow sub-national county Ministries of Health to tailor existing national recommendations for control. Future research should aim at relating these heterogeneities in TPR with traditional community-level prevalence to improve micro-stratification or and at granular and specific levels to improve our ability to track the impact of vaccination interventions targeted for young children below 2 years.

## Supplementary Information

**Additional file 1.** Population modelling.

**Additional file 2.** Statistical methodology for TPR modelling.

**Additional file 3.** Extended results for TPR estimate by sub-county.

## Data Availability

Aggregated DHIS2 data is available online with access provided by Ministry of Health https://hiskenya.org/dhis-web-commons/security/login.action. The datasets used and/or analysed during the current study are also available from the corresponding author on reasonable request.

## Abbreviations

DHIS2: District Health Information System 2
EA: Enumeration area
EP: Exceedance probability
GTS: Global Technical Strategy
HB-HI: High burden-high impact
INLA: Integrated nested Laplace approximation
IRS: Indoor residual spraying
LLIN: Long-lasting insecticide-treated nets
MAE: Mean absolute error
MOH: Ministry of Health
MPE: Mean prediction error
NMCP: National malaria control programmes
RDT: Rapid diagnostic test
RF: Random forest
RMSE: Root mean square error
SPDE: Stochastic partial differential equation
SSA: Sub-Saharan Africa
TPR: Test positivity rate
WHO: World Health Organization
