# Supplementary material for: Malaria micro-stratification using routine surveillance data in Western Kenya
Source: Malar J. 2021 Jan 7;20:22. doi: 10.1186/s12936-020-03529-6 (PMC7788718; doi:10.1186/s12936-020-03529-6)
Supplement: Supplementary file 1 — Additional file 1. Population modelling. [file 12936_2020_3529_MOESM1_ESM.docx]

**Additional Information 1: Population modelling**

***Machine learning informed population disaggregation at 100m grids***

Population disaggregation was conducted using a random forest-informed dasymetric redistribution technique [1-3]. The disaggregation uses weights that capture the underlying heterogeneous distribution of population within the input polygon.

A Random Forest consists of a set of classification and regression treeswhere is the input vector, are independent and identically distributed random vectors generated for thetree based on a training set of data [4]. A fundamental property of the random forest algorithm is that as the number of regression trees increases, out-of-bag (OOB) error, calculated using hold-out data produced during random sampling with replacement, reduces significantly with large sample size [4-7]. Here, after excluding areas with zero population counts such as lakes or national parks and game reserves, each regression tree was trained on the log-transformed EA population density and the EA level covariate summaries (Table S2) [4]. Then the average prediction of the back-transformed population densities, from all trees, was produced using the pixel-level covariate values and these estimates were used as the weighting layer in the dasymetric disaggregation of population counts to 100m x 100m pixels. The disaggregated pixel-level (target areas) population counts add up to the total of the EA unit from which they were disaggregated from [1, 3].

The population sizes at the national level for each dataset were projected to 2018 and 2019 using the equation below

, where is the new population projection within a pixel, is the population within the same pixel at the year of the input population data, is the number of years between the input data and new estimate, and is the mean inter-census growth rate.

**Table S1**: Metadata description for covariates used in population modelling

| **Covariate** | **Description** |
| --- | --- |
| Land cover^1^ | Globcover is usually classified into 22 classes similar to the United Nation’s Land Cover Classification System (UN-LCCS)[8]. The current GlobCover V.2.3 is derived from a time-series of Medium Resolution Imaging Spectrometer (MERIS) satellite imagery acquired from December 2004 to June 2006 at a spatial resolution of 300 meters.  Here, the final covariated derived covariates from land cover classes: cls011& dst011 (cropland), cls040 &dst040 (forest), dst130 (Shrubland), cls140 & dst140 (grassland), dst150 (sparse vegetation), dst160 (regulary flooded broadleaved forest), cls190 (artifictial/urban areas), dst200 (bare areas), dst210 (major water bodies), dstBLT (built-up-areas) |
| Globe Cover satellite imagery, raster spatial resolution 300m Resampled to 100m and reclassified, refined with settlements extents [9] |
| Night-time lights | Suomi NPP VIIRS-Derived 2012 Lights at Night, 15 arc-second. Lights at night from Visible Infrared Imaging Radiometer (VIIR) sensor 2012 |
| Elevation | [DEM, HydroSHEDS void-filled (Lehnert, et al., 2006), http://hydrosheds.cr.usgs.gov/dataavail.php](http://hydrosheds.cr.usgs.gov/dataavail.php) [10] |
| Water bodies | Rivers and inland water bodies data downloaded from the National Geospatial-Intelligence Agency (NGA). Originally VMAP0 data sources provided by the NGA |
| Protected areas or parks | World database on protected areas. Description of protected areas a joint project between the United Nations Environment Programme (UNEP) and the International Union for Conservation of Nature (IUCN), managed by UNEP World Conservation Monitoring Centre (UNEP-WCMC) [11] |
| Built areas | Derived from approaches to settlement mapping for public health management in Kenya using medium spatial resolution satellite imagery [12]. |

**Figure S1: Modelled population map for Western Kenya for 2019.**

As an indicator of predictive performance, the proportion of variance explained by RF modelling was 75%%. The out-of-bag error rate as measured by the mean square error was 0.97.

***Adjusting population to match Kenya 2019 census***

The projected modelled population maps were adjusted to the most recent census up to Administrative level 2 (the sub-county). A sum of population from the modelled map was obtained using ArcGIS spatial analytical tools. The modelled total count was then compared to the official census data to compute an adjustment factor for each sub-county polygon. The adjustment factor was then re-applied to the continuous 100m pixels to obtain an adjusted 2019 census map.

**References**

1. Mennis J, Hultgren T: **Intelligent Dasymetric Mapping and Its Application to Areal Interpolation.** *Cartography and Geographic Information Science* 2006, **33:**179-194.

2. Mennis J: **Generating Surface Models of Population Using Dasymetric Mapping*.** *The Professional Geographer* 2003, **55:**31-42.

3. Stevens FR, Gaughan AE, Linard C, Tatem AJ: **Disaggregating census data for population mapping using Random Forests with remotely-sensed and ancillary data.** *PLoS One* 2015, **10:**e0107042.

4. Breiman L: **Random Forests.** *Machine Learning* 2001, **45:**5-32.

5. Janitza S, Hornung R: **On the overestimation of random forest’s out-of-bag error.** *PLOS ONE* 2018, **13:**e0201904.

6. Mitchell MW: **Bias of the Random Forest Out-of-Bag (OOB) Error for Certain Input Parameters.** *Open Journal of Statistics* 2011, **Vol.01No.03:**7.

7. Breiman L: **Bagging Predictors.** *Machine Learning* 1996, **24:**123-140.

8. **Land Cover Classification System (LCCS): Classification Concepts and User Manual** [ <http://www.fao.org/docrep/003/x0596e/x0596e00.htm> ] Accessed: *accessed October 2018*

9. Arino O, Gross D, Ranera F, Bourg L, Leroy M, Bicheron P, Latham J, Di Gregorio A, Brockman C, Witt R, et al: **GlobCover: ESA service for global land cover from MERIS.** In *Proceedings of the International Geoscience and Remote Sensing Symposium (IGARSS) 2007.* Barcelona: IEEE International; 2007: 2412 - 2415

10. Lehner B, Verdin K, Jarvis A: **New Global Hydrography Derived From Spaceborne Elevation Data.** *Eos, Transactions American Geophysical Union* 2008, **89:**93-94.

11. **World database on protected areas** [<https://www.iucn.org/theme/protected-areas/>] Accessed: *December, 2019*

12. Tatem AJ, Noor AM, Hay SI: **Defining approaches to settlement mapping for public health management in Kenya using medium spatial resolution satellite imagery.** *Remote Sensing of Environment* 2004, **93:**42-52.
