# Supplementary material for: Malaria micro-stratification using routine surveillance data in Western Kenya
Source: Malar J. 2021 Jan 7;20:22. doi: 10.1186/s12936-020-03529-6 (PMC7788718; doi:10.1186/s12936-020-03529-6)
Supplement: Supplementary file 2 — Additional file 2. Statistical methodology for TPR modelling. [file 12936_2020_3529_MOESM2_ESM.docx]

**Additional file 2**

Contents

[1.0 GATHER Checklist 2](#_Toc54957836)

[2.0 Exploratory analysis of TPR 3](#_Toc54957837)

[2.1 Comparison of crude TPR by health facility type 3](#_Toc54957838)

[2.2 Crude maps of TPR 4](#_Toc54957839)

[2.3 Health facilities reporting less than 12 months 5](#_Toc54957840)

[2.4 Temporal plot of malaria cases in western Kenya 5](#_Toc54957841)

[2.5 Justification of using a spatial analysis approach 5](#_Toc54957842)

[3.0 Use of SPDE approach in R-INLA for TPR prediction 6](#_Toc54957843)

[3.1 SPDE space-time code 7](#_Toc54957844)

[3.2 Mesh Construction 7](#_Toc54957845)

[3.3 Continuous predicted TPR Maps 8](#_Toc54957846)

[2.3.1 Model fitting results 9](#_Toc54957847)

[3.3.2 Validation plots 9](#_Toc54957848)

# 1.0 GATHER Checklist

**Table S1.** Guidelines for Accurate and Transparent Health Estimates Reporting (GATHER) of information that should be included in reports of global health estimates.

| **Item #** | **Description** | **Reported on page #** |
| --- | --- | --- |
| **Objectives and Funding** | | |
| **1** | Define the indicator(s), populations (including age, sex, and geographic entities), and time period(s) for which estimates were made. | Main Paper: Background & Methods sections |
| **2** | List the funding sources for the work. | Main Paper: Acknowledgements/Funding statement |
| **Data Inputs** | | |
| *For all data inputs from multiple sources that are synthesized as part of the study:* | | |
| **3** | Describe how the data were identified and how the data were accessed. | Main Paper: Methods (Routine malaria data from DHIS2, Population) |
| **4** | Specify the inclusion and exclusion criteria. Identify all ad-hoc exclusions. | Main Paper: Methods, (Data preprocessing) |
| **5** | Provide information on all included data sources and their main characteristics. For each data source used, report reference information or contact name/institution, population represented, data collection method, year(s) of data collection, sex and age range, diagnostic criteria or measurement method, and sample size, as relevant. | Main Paper: DHIS 2 Routine data |
| **6** | Identify and describe any categories of input data that have potentially important biases (e.g., based on characteristics listed in item 5). | Main Paper: Results & Discussion (Limitations e.g. Age reporting, missing data reports) |
| *For data inputs that contribute to the analysis but were not synthesized as part of the study:* | | |
| **7** | Describe and give sources for any other data inputs. | Supplementary Information 2.0: Description of population data |
| *For all data inputs:* | | |
| **8** | Provide all data inputs in a file format from which data can be efficiently extracted (e.g., a spreadsheet rather than a PDF), including all relevant meta-data listed in item 5. For any data inputs that cannot be shared because of ethical or legal reasons, such as third-party ownership, provide a contact name or the name of the institution that retains the right to the data. | DHIS2 data aggregate data publically available. Login request to the Ministry of Health can be obtained online  (<https://hiskenya.org/dhis-web-commons/security/login.action>) |
| **9** | Provide a conceptual overview of the data analysis method. A diagram may be helpful. | Main paper: Methods (Data),  Additional methods in the  Figure 2 main paper |
| **10** | Provide a detailed description of all steps of the analysis, including mathematical formulae. This description should cover, as relevant, data cleaning, data pre-processing, data adjustments and weighting of data sources, and mathematical or statistical model(s). | Main Paper: Methods (Space-time geostatistical analysis of TPR), Supplementary Information 2: The use of SPDE approach |
| **11** | Describe how candidate models were evaluated and how the final model(s) were selected. | Main Paper: Methods (Model validation), Supplementary Information 2 (Model validation) |
| **12** | Provide the results of an evaluation of model performance, if done, as well as the results of any relevant sensitivity analysis. | Main Paper: Results (Model sensitivity analysis results), Supplementary Information 2: Model validation results |
| **13** | Describe methods for calculating uncertainty of the estimates. State which sources of uncertainty were, and were not, accounted for in the uncertainty analysis. | Main Paper: Methods (Description of space-time modelling, Posterior distribution and using exceedance probability (uncertainty) for micro-stratification. |
| **14** | State how analytic or statistical source code used to generate estimates can be accessed. | Supplementary information 2: SPDE space time code |
| **Results and Discussion** | | |
| **15** | Provide published estimates in a file format from which data can be efficiently extracted. | Main Paper: Raster files for spatial data in supplementary information to be deposited online (link to be provided) |
| **16** | Report a quantitative measure of the uncertainty of the estimates (e.g., uncertainty intervals). | Main Paper: Results 95% Bayesian credible intervals reported. |
| **17** | Interpret results in light of existing evidence. If updating a previous set of estimates, describe the reasons for changes in estimates. | Main Paper: Results and discussion |
| **18** | Discuss limitations of the estimates. Include a discussion of any modelling assumptions or data limitations that affect interpretation of the estimates. | Main Paper: Discussion |

# 2.0 Exploratory analysis of TPR

## 2.1 Comparison of crude TPR by health facility type

Figure S1: Box plot showing TPR by facility type (A) dispensaries, health centres, and hospitals and (B) Public and Private. The hospital TPR rate was lower, possibly due to tendency to use of microscopy rather than Rapid Diagnostic Test (RDTs). RDT use at primary care facilities detect parasite antigen. There was little difference in TPR between public when compared to private based facilities.

## 2.2 Crude maps of TPR

Figure S2: Aggregated data of Crude maps of TPR over the 2 years **A)** Facility-level average positivity rate and B) Sub-county level average.

## 2.3 Health facilities reporting less than 12 months

(***n*=263**)


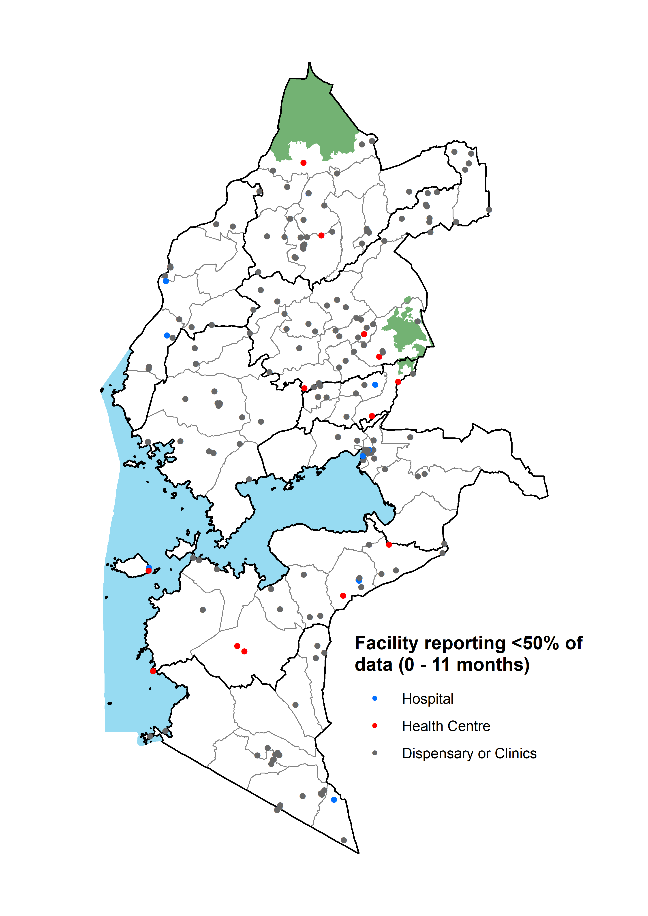


## 2.4 Temporal plot of malaria cases in western Kenya

Figure S3: Box plot of confirmed malaria cases over the 24-month period.

## 2.5 Justification of using a spatial analysis approach

Preliminary analysis to establish the validity of using a modelling structure that accounts for spatial dependencies in the data was explored using semi-variogram [1, 2]. Semi-variograms summarise spatial dependencies in the data.


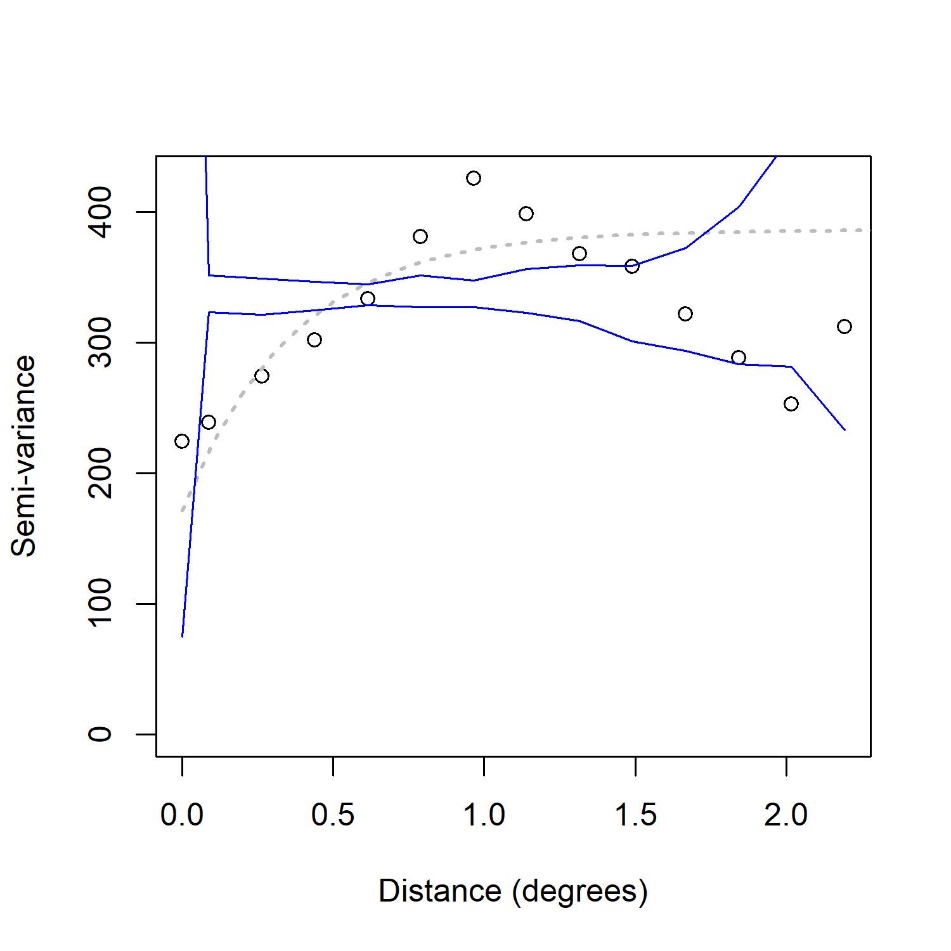


Figure S4: Spatial exploratory semi-variogram plot for data. The y-axis is half variance between pairs of data points while the x-axis in the distance. The blue lines represent the variogram envelopes encompassing the 95% confidence interval after 10000 monte-carlo simulation. Since a considerable part of the variogram plot lied outside of these margins suggested a presence of spatial structure that could not be ignored.

# 3.0 Use of SPDE approach in R-INLA for TPR prediction

We begin by developing the likelihood under the linear predictor. Let $\mu_{t}=X_{t}\beta(t)$then,

$$Y=\mu+\alpha\otimes1_{nx1}+1_{Tx1}\otimes w+e$$

Where denotes the Kronecker product. Thus given all the parameters.

$$Y|\beta,\alpha,w,\sigma_{e}^{2}\sim N\left(\mu+\alpha\otimes1_{nx1}+1_{Tx1}\otimes w,\sigma_{e}^{2}I_{T_{n}xT_{n}}\right)$$

with $w\sim GP(0,\Sigma_{w})$and $\alpha\left(t\right)$follow a random walk. The likelihood arises as a product of independent normal densities. Model was implemented in R-INLA [3]. R-INLA uses both analytical approximation and numerical integration to perform approximate Bayesian inference for the class of latent Gaussian models, such as the spatio-temporal models. The geostatistical implementation in R-INLA is implemented via the space-time stochastic partial differential equation (SPDE) approach [4, 5]

$$\frac{\partial}{\partial t}\left(k^{2}-\Delta\right)^{\alpha/2}\left(\tau x(s,t)\right)=w(s,t)$$

Where $\left(k^{2}-\Delta\right)^{\alpha/2}$ is a differential operator, k is a scaling parameter, $\Delta$ is the Laplacian, $\alpha$controls the smoothness of realizations, $\tau$the variance and $x(s,t)$is the spatial-temporal domain for s(s_1_,……,s_n_) locations. $w(s,t)$is the Gaussian white noise. The space time SPDE generates a precision matrix for the weight vector x as $Q=Q_{t}\otimes Q_{s}$Qs, where Qs is the precision for the previous purely spatial model, and Qt is the precision corresponding to a one-dimensional random walk.

The link between Matérn smoothness $\nu$and variance $\sigma^{2}$is given by $\alpha=\nu+d/2$where *d* is spatial dimension and marginal variance$\sigma^{2}=1/\left(\Gamma(\nu)\Gamma(\alpha)(4\pi)^{d/2}k^{2\nu}\tau^{2}\right)$. The above SPDE is solved via finite element method by triangulating the domain of interest. The SPDE is projected to a basis representation,

$$x(s,t)=\sum_{k} \psi_{k}(s,t)x_{k}$$

Where $\psi_{k}(s,t)=\psi_{i}^{s}(s)\cdot\psi_{j}^{t}(t)$a product of spatial and temporal basis function

Several advantages arise from such construction. First, the aforementioned resulting sparse covariance matrix is computationally efficient since GMRF is only discretely indexed at locations *x*_1_,……*x*_k_. Secondly the covariance function is flexible within the general family of covariances. The stationary Matérn covariance function for spatial lag distance$h=\left\|s_{1}-s_{2}\right\|$, s_1_ and s_2_ locations is expressed as:

$$C(s_{1}s_{2})=\frac{\sigma^{2}}{2^{v-1}\Gamma(\nu)}\left(kh\right)^{\nu}k_{\nu}\left(kh\right)$$

Where $k_{\nu}$ is the modified Bessel function of second order and $h$ is the Euclidean distance while $\sigma^{2}$is the marginal variance.

## 3.1 SPDE space-time code

prec.prior **<-** list**(**theta**=**list**(**prior **=** 'pc.prec', param **=** c**(**1, 0.01**)))**

h.spec **<-** list**(**theta **=** list**(**prior **=** 'pccor1', param **=** c**(**0.7, 0.7**)))**

#

formula **<-** hospconf **~** **-**1 **+** Intercept +

as.factor**(**year**)** **+**

as.factor**(**hftype**)+**

f**(**time, model **=** 'rw2', scale.model **=** **TRUE**,

hyper **=** list**(**theta **=** list**(**prior**=**'pc.prec', param**=**c**(**1,0.01**)))** **)** **+**

f**(**monthly, model **=** 'rw2', scale.model **=** **TRUE** ,

hyper **=** prec.prior **)** **+**

f**(**i, model**=**spde1, group **=** i.group, control.group **=** list**(**model**=**"ar1", hyper**=** h.spec**)** **)**

Spacetime.Model **<-** inla**(**formula,

data**=**inla.stack.data**(**stack.hosp**)**,

family**=**'binomial',

Ntrials**=**Ntrials,

control.inla**=**list**(**strategy**=**"laplace", tolerance **=** 1e**-**20, h**=**1e**-**05**)**,

control.predictor**=**list**(**A**=**inla.stack.A**(**stack.hosp**)**,

compute**=TRUE**,

link**=**1**)**,

quantiles **=** c**(**0.025, 0.05,0.5,0.95, 0.975**)**,

control.fixed**=** list**(**expand.factor.strategy **=** "inla",

mean.intercept **=** 0**)**,

control.compute**=**list**(**config**=TRUE**, cpo**=TRUE**, dic**=TRUE**, mlik**=TRUE)**,

verbose**=TRUE)**

save.image**(**file**=**'hosptpr.RData'**)**

## 3.2 Mesh Construction

Continuous spatial random effects was modelled using stochastic partial differential equations (SPDE) representations of Gaussian-Markov random field (GMRF) [6] approximations of a spatially-autocorrelated Gaussian process, using triangular finite element meshes as implemented in the R-INLA R package. Minimum and maximum edge lengths were set to 5- and 20-km. A 10-km external buffer was used to avoid edge effects. Spatial meshes for the region are shown in in Supplementary Figure S5.


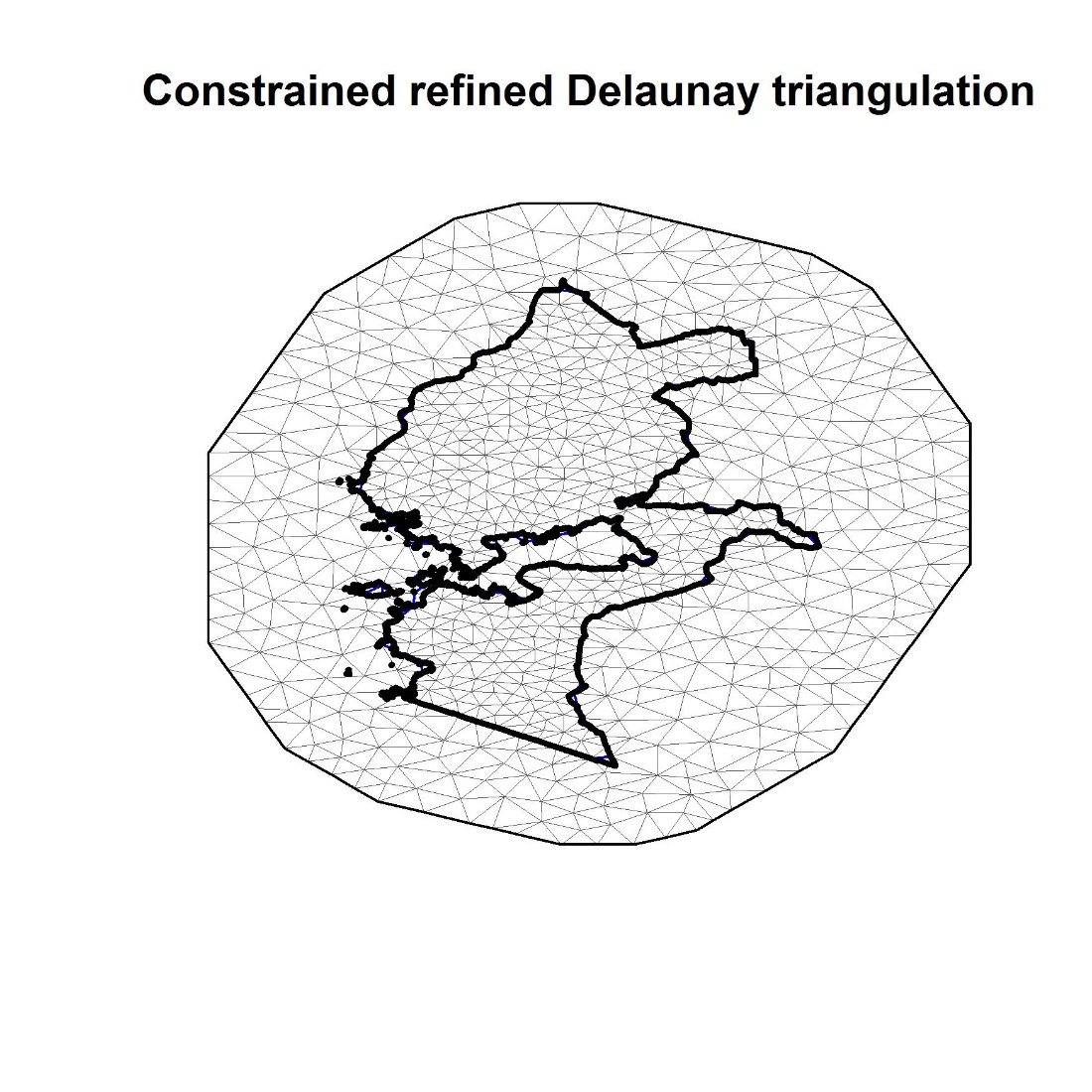


Figure S5: Mesh construction for the 8 counties in Western Kenya

## 3.3 Continuous predicted TPR Maps

Estimated mean TPR and uncertainty (range of upper and lower 95%) are plotted jointly at 1 × 1-km spatial resolution (Supplementary Figures x).

Figure S6: Bayesian predictions at 1 x 1 km of A) Posterior mean continuous maps of TPR and B) uncertainty map of the difference in upper and lower 95% credible intervals.


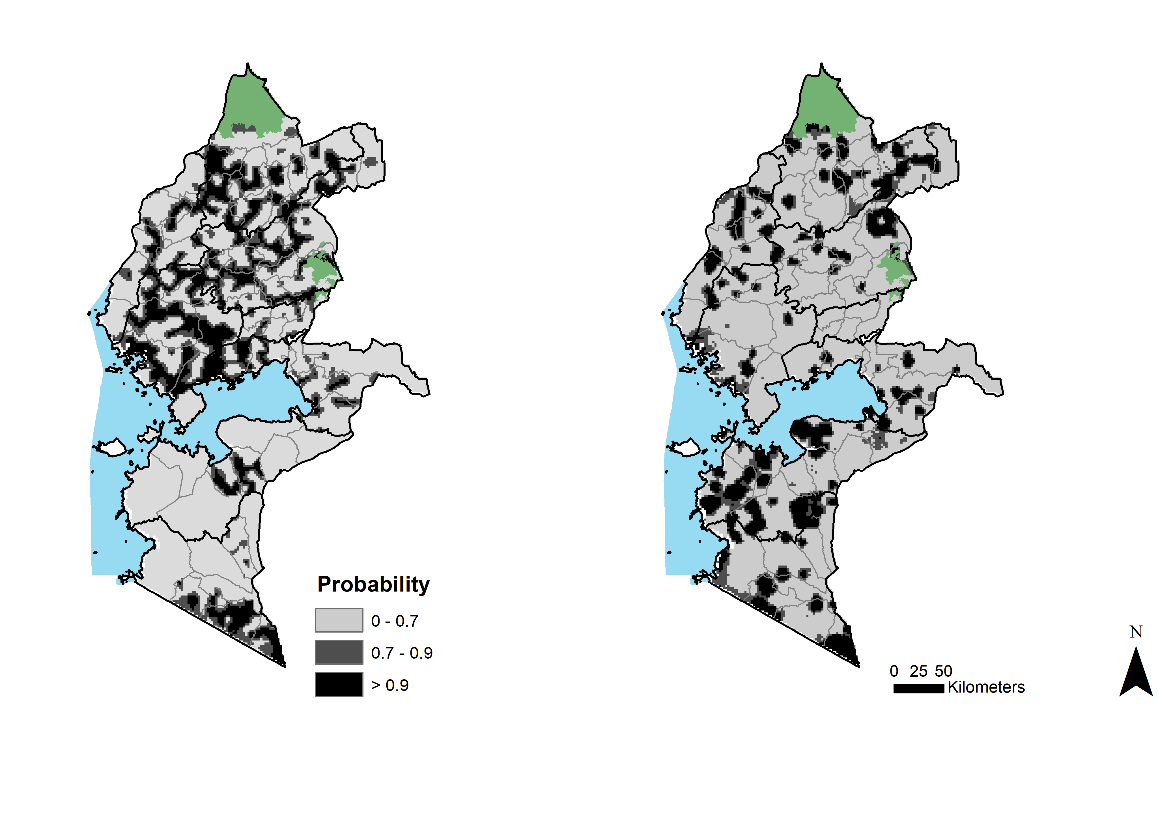


Figure S7: Bayesian predictions at 1 x 1 km of A) Probability of exceeding 70% TPR binned continuous map and B) Probability of TPR <30% binned continuous map.

### 2.3.1 Model fitting results

Model parameter estimates from regional MBG models are summarized in Supplementary Table S2. Spatial range is the distance at which spatial autocorrelation has declined to approximately 0.1.

Table S2: Bayesian model parameter estimates.

| **Parameter** | **Mean (95% Bayesian Credible Interval)** |
| --- | --- |
| Year (2018) | - |
| Year (2019) | 0.425 (0.425 - 0.433) |
| Disp | - |
| HC | 2.170 (2.163 - 2.190) |
| Hosp | 1.974 (1.968 - 1.991) |
| Spatial range | 0.186 (0.184 - 0.243) |
| Marginal variance | 4.524 (3.966 - 5.176) |
| ARI (Rho) | 0.763 (0.765 - 0.837) |
| Precision for the Month | 0.056 (0.025 - 0.108) |

### 3.3.2 Validation plots

Figure S8: Validation plots showing **A**) Scatter plot of the association between the observed against predictions of the 20% (n=360) subset data. Pearson correlation 64%. **B**) the Q-Q plot that shows quantiles of residuals compared to quantiles of normal distribution. This plot depicts any deviation of residual from the normal distribution. **C)** Semi-variogram of residuals showing minimal spatial structure after modelling,

**References**

1. Cressie N, Johannesson G: **Fixed rank kriging for very large spatial data sets.** *Journal of the Royal Statistical Society: Series B (Statistical Methodology)* 2008, **70:**209-226.

2. Cressie N: *Statistics for spatial data.* 2 edn. New York: Wiley; 1993.

3. Rue H, Martino S, Chopin N: **Approximate Bayesian inference for latent Gaussian models by using integrated nested Laplace approximations.** *Journal of the Royal Statistical Society: Series B (Statistical Methodology)* 2009, **71:**319-392.

4. Lindgren F: **Continuous domain spatial models in R-INLA.** *ISBA Bulletin* 2013, **19:**14-20.

5. Lindgren F, Rue H, Lindström J: **An explicit link between Gaussian fields and Gaussian Markov random fields: the stochastic partial differential equation approach.** *Journal of the Royal Statistical Society: Series B (Statistical Methodology)* 2011, **73:**423-498.

6. Rue H, Held L: *Gaussian Markov Random Fields: Theory and Applications (Chapman & Hall/CRC Monographs on Statistics & Applied Probability).* Chapman and Hall/CRC; 2005.
