# Supplementary material for: Malaria micro-stratification using routine surveillance data in Western Kenya
Source: Malar J. 2021 Jan 7;20:22. doi: 10.1186/s12936-020-03529-6 (PMC7788718; doi:10.1186/s12936-020-03529-6)
Supplement: Supplementary file 3 — Additional file 3. Extended results for TPR estimate by sub-county. [file 12936_2020_3529_MOESM3_ESM.docx]

**Additiona file 3**

**Figure S1: Coded map of sub-counties in Western Kenya**

**Table S1**: showing summary of health facility data in the 64 sub-counties, the predicted mean test positivity rate (95% Bayesian credible interval) and the stratification category based on exceedance probability. Stratification classification: class 1 (<30%); class 2 (>30% -< 40%); class 3 (>40 -< 70); class 4 (> 70%).

| County | Sub-county | Sub-county  ID | Sub-County Pop 2019 | Number of health facilities | Tested | Out-patient confirmed malaria cases | Predicted test positivity rate mean (Bayesian 95% credible interval) | TPR stratification category |
| --- | --- | --- | --- | --- | --- | --- | --- | --- |
| Bungoma | Bumula | 1 | 215,892 | 17 | 200,194 | 108,223 | 62 (60.6 - 63.3) | 4 |
| Bungoma | Kabuchai | 2 | 177,748 | 26 | 169,698 | 86,098 | 57.3 (56.3 - 58.3) | 2 |
| Bungoma | Kanduyi | 3 | 287,765 | 48 | 432,175 | 156,889 | 57.3 (56.2 - 58.5) | 3 |
| Bungoma | Kimilili | 4 | 162,038 | 21 | 160,421 | 54,526 | 53.3 (52.2 - 54.4) | 3 |
| Bungoma | Mt Elgon | 5 | 218,529 | 38 | 192,920 | 77,542 | 38.7 (17.9 - 72.2) | 1 |
| Bungoma | Sirisia | 6 | 119,875 | 19 | 236,863 | 101,747 | 68.4 (67.4 - 69.5) | 4 |
| Bungoma | Tongaren | 7 | 221,660 | 24 | 117,953 | 50,734 | 50.5 (48.3 - 52.7) | 3 |
| Bungoma | Webuye East | 8 | 114,548 | 24 | 120,667 | 59,339 | 55.1 (54.1 - 56.2) | 3 |
| Bungoma | Webuye West | 9 | 152,515 | 19 | 219,052 | 85,513 | 63 (62 - 64) | 3 |
| Busia | Bunyala | 10 | 85,977 | 10 | 128,286 | 80,931 | 71.7 (68.2 - 75.1) | 3 |
| Busia | Butula | 11 | 140,334 | 22 | 219,639 | 169,568 | 77.7 (76.9 - 78.6) | 4 |
| Busia | Samia | 12 | 107,176 | 18 | 150,817 | 98,048 | 73.1 (68.5 - 77.3) | 4 |
| Busia | Matayos | 13 | 142,408 | 32 | 328,329 | 166,223 | 71.9 (71 - 72.9) | 4 |
| Busia | Nambale | 14 | 111,636 | 12 | 143,328 | 99,275 | 60.9 (59.4 - 62.4) | 3 |
| Busia | Teso North | 15 | 138,034 | 23 | 249,241 | 142,688 | 63.7 (61.5 - 65.8) | 3 |
| Busia | Teso South | 16 | 168,116 | 22 | 225,532 | 136,703 | 74.9 (70.9 - 77.7) | 4 |
| Homa Bay | Homa Bay | 17 | 117,439 | 31 | 124,180 | 34,521 | 39.5 (37.7 - 41.3) | 2 |
| Homa Bay | Ndhiwa | 18 | 218,136 | 45 | 152,929 | 55,709 | 35.7 (33.1 - 38.4) | 1 |
| Homa Bay | Rachuonyo East | 19 | 121,822 | 30 | 96,900 | 29,241 | 23.6 (22.1 - 25.1) | 1 |
| Homa Bay | Rachuonyo North | 20 | 178,686 | 42 | 163,309 | 53,440 | 30.5 (28.5 - 32.6) | 1 |
| Homa Bay | Rachuonyo South | 21 | 130,814 | 37 | 171,549 | 53,667 | 30.1 (28.5 - 31.8) | 1 |
| Homa Bay | Rangwe | 22 | 117,732 | 29 | 124,482 | 50,189 | 39.2 (36.9 - 41.5) | 2 |
| Homa Bay | Suba North | 23 | 124,938 | 44 | 119,734 | 40,297 | 34.4 (28.6 - 40.3) | 1 |
| Homa Bay | Suba South | 24 | 122,383 | 34 | 71,928 | 20,291 | 32.4 (28.3 - 37) | 1 |
| Kakamega | Butere | 25 | 154,100 | 23 | 293,712 | 191,638 | 70.2 (69.6 - 70.7) | 4 |
| Kakamega | Ikolomani | 26 | 111,743 | 27 | 184,701 | 80,287 | 56.1 (55.3 - 56.9) | 4 |
| Kakamega | Khwisero | 27 | 113,476 | 25 | 306,453 | 161,186 | 65 (64.4 - 65.6) | 4 |
| Kakamega | Likuyani | 28 | 152,055 | 28 | 56,583 | 11,752 | 40 (31 - 47.5) | 1 |
| Kakamega | Lugari | 29 | 188,900 | 36 | 156,393 | 43,620 | 42.1 (40.1 - 44) | 1 |
| Kakamega | Lurambi | 30 | 188,212 | 37 | 409,832 | 192,102 | 58 (57.3 - 58.8) | 4 |
| Kakamega | Malava | 31 | 238,330 | 27 | 262,973 | 130,185 | 53.7 (52 - 55.4) | 2 |
| Kakamega | Matungu | 32 | 166,940 | 20 | 228,677 | 147,104 | 64.1 (63 - 65.3) | 3 |
| Kakamega | Mumias East | 33 | 116,851 | 19 | 225,573 | 121,006 | 63.3 (62.4 - 64.1) | 4 |
| Kakamega | Mumias West | 34 | 115,354 | 18 | 229,698 | 105,762 | 66 (65.2 - 66.9) | 3 |
| Kakamega | Navakholo | 35 | 153,977 | 18 | 225,236 | 121,963 | 59.1 (58.1 - 60.2) | 4 |
| Kakamega | Shinyalu | 36 | 167,641 | 38 | 199,866 | 76,525 | 52.4 (48.2 - 56.1) | 2 |
| Kisumu | Kisumu Central | 37 | 174,145 | 55 | 288,164 | 71,462 | 42.9 (42 - 43.9) | 2 |
| Kisumu | Kisumu East | 38 | 220,997 | 28 | 190,825 | 88,339 | 54.5 (53.2 - 55.8) | 4 |
| Kisumu | Kisumu West | 39 | 172,821 | 31 | 236,220 | 109,781 | 53.3 (52.3 - 54.4) | 3 |
| Kisumu | Muhoroni | 40 | 154,116 | 34 | 236,293 | 106,581 | 49 (35.8 - 64.6) | 1 |
| Kisumu | Nyakach | 41 | 150,320 | 30 | 203,959 | 83,943 | 51.2 (49.5 - 52.9) | 1 |
| Kisumu | Nyando | 42 | 161,508 | 29 | 244,555 | 124,676 | 54.1 (52.4 - 55.9) | 3 |
| Kisumu | Seme | 43 | 121,667 | 25 | 217,148 | 122,239 | 60.6 (59.3 - 61.9) | 4 |
| Migori | Awendo | 44 | 117,290 | 29 | 98,111 | 30,716 | 37.1 (35.2 - 39) | 2 |
| Migori | Kuria East | 45 | 96,872 | 31 | 190,288 | 101,654 | 60.8 (59 - 62.6) | 4 |
| Migori | Kuria West | 46 | 208,513 | 48 | 332,125 | 157,359 | 54.5 (52.7 - 56.3) | 3 |
| Migori | Nyatike | 47 | 176,162 | 47 | 187,670 | 73,679 | 40.7 (36.3 - 45.5) | 1 |
| Migori | Rongo | 48 | 124,587 | 22 | 105,228 | 24,767 | 31.3 (29.6 - 33.1) | 1 |
| Migori | Suna East | 49 | 122,674 | 17 | 116,554 | 26,802 | 40.4 (38.3 - 42.4) | 3 |
| Migori | Suna West | 50 | 128,890 | 34 | 143,596 | 54,686 | 46.4 (43.8 - 49.1) | 3 |
| Migori | Uriri | 51 | 141,448 | 26 | 120,621 | 38,911 | 41.8 (40 - 43.7) | 1 |
| Siaya | Alego Usonga | 52 | 224,343 | 33 | 285,363 | 177,260 | 66.2 (65.1 - 67.3) | 4 |
| Siaya | Bondo | 53 | 197,883 | 60 | 366,474 | 161,425 | 55.8 (52.8 - 58.6) | 3 |
| Siaya | Gem | 54 | 179,792 | 46 | 479,986 | 265,836 | 67.3 (66.4 - 68.1) | 4 |
| Siaya | Rarieda | 55 | 152,570 | 45 | 348,343 | 172,888 | 54.7 (51.9 - 57.7) | 3 |
| Siaya | Ugenya | 56 | 134,354 | 26 | 268,101 | 151,805 | 62.9 (61.5 - 64.4) | 4 |
| Siaya | Ugunja | 57 | 104,241 | 22 | 261,373 | 140,452 | 61.4 (60.6 - 62.3) | 4 |
| Vihiga | Emuhaya | 58 | 97,141 | 4 | 21,021 | 13,598 | 58.2 (57.5 - 58.8) | 4 |
| Vihiga | Hamisi | 59 | 159,258 | 34 | 240,967 | 115,983 | 54.9 (53.6 - 56.1) | 2 |
| Vihiga | Luanda | 60 | 106,694 | 21 | 176,983 | 79,912 | 55.8 (55 - 56.5) | 4 |
| Vihiga | Sabatia | 61 | 131,628 | 24 | 120,593 | 52,830 | 41.6 (40.6 - 42.6) | 2 |
| Vihiga | Vihiga | 62 | 95,292 | 20 | 202,671 | 72,046 | 53.5 (52.8 - 54.3) | 3 |
